# Higher expression of calcineurin predicts poor prognosis in unique subtype of ovarian cancer

**DOI:** 10.1186/s13048-019-0550-0

**Published:** 2019-08-09

**Authors:** Bing Xin, Kai-Qiang Ji, Yi-Si Liu, Xiao-Dong Zhao

**Affiliations:** 1 Department of Gynaecology, Shengjing Hospital of China Medical University, No.36 Sanhao Street, Heping District, Shenyang, 110004 China; 2 Department of ICU, Shengjing Hospital of China Medical University, Shenyang, 110004 China; 3 Department of Pathology, Shengjing Hospital of China Medical University, Shenyang, 110004 China

**Keywords:** Calcineurin, Prognosis, Histological subtype, Ovarian cancer, Carcinoembryonic antigen, Cancer antigen 72–4

## Abstract

**Background:**

The role of calcineurin/NFAT signaling in ovarian cancer has been unknown. NFAT was significantly overexpressed in ovarian cancer tissues and that overexpression of NFAT was significantly associated with metastasis and poor prognosis on clinical tissue level. To investigate whether NFAT upstream protein, calcineurin (CN), affects the prognosis in various histological subtype of ovarian cancer (OC).

**Methods:**

The association between CN and clinical features was analyzed in 50 OC patients treated from 2007 to 2012. CN expression was examined using immunohistochemistry. We observed the association of CN expression with the prognosis in these patients.

**Results:**

CN expression was significantly increased in later-stage tumor tissue of serous carcinoma compared with those with early-stage. The expression of CN positively correlated with the serum cancer antigen 125 (CA125) level in ovarian clear-cell carcinoma and the serum alpha-fetoprotein (AFP) level in papillary serous cystadenocarcinoma. Particularly, higher CN expression in tumor tissues significantly correlated with reduced overall survival among patients with serous carcinoma. In addition, the serum cancer antigen 72–4 (CA72–4) level, serum carcinoembryonic antigen (CEA) levels, pathological stage, lymph node metastasis, and chemotherapeutic resistance were identified as significant prognostic factors in ovarian clear-cell carcinoma, serous carcinoma, or papillary serous cystadenocarcinoma.

**Conclusions:**

CN is upregulated in ovarian cancer tissues with later-stage and that the expression of CN, CA72–4, and CEA was remarkably associated with poor prognosis in unique subtype of ovarian cancer. CN levels may be investigated for use as a prognostic biomarker for risk assessment in unique subtype of OC patients.

**Electronic supplementary material:**

The online version of this article (10.1186/s13048-019-0550-0) contains supplementary material, which is available to authorized users.

## Background

Ovarian cancer (OC) is the leading cause of death from gynecological malignancies, accounting for 4% of all cancers in women in the worldwide [1]. Ovarian cancers can be classified into three large groups: epithelial, germ cell, and specialized stromal cell tumors. The vast majority of ovarian cancers are epithelial ovarian cancers (EOCs). EOC can be further subdivided into various histological subtypes that fall into two main groups: Type I and Type II tumors. Type I tumors include low-grade serous, mucinous, endometrioid, clear cell carcinomas and tend to grow more slowly, often from an identifiable precursor. In contrast, Type II tumors are characterized by high-grade and rapidly progressive disease. High-grade serous ovarian carcinoma and papillary serous cystadenocarcinomas are the most common Type II tumor. Unfortunately, it is also one of the most aggressive. Patients with stage III or IV disease have a dismal 25% 5-year survival rate [2]. In spite of improvements of treatment, survival rates for patients with advanced disease remain gloomy [3]. However, the molecular mechanism of ovarian cancer initiation and progression has been still poorly understood. Therefore, it is essential to understand its molecular mechanism before establishing novel therapeutic and diagnostic strategies against the deadly disease.

Calcineurin (CN) is composed of two subunits, a catalytic subunit called calcineurin A (CNA) encoded by three separate genes (PPP3CA, PPP3CB and PPP3CC), and a regulatory subunit, calcineurin B (CNB) encoded by two genes, PPP3R1 and PPP3R2, with the latter restricted to testis and brain. CN is a calcium dependent serine/threonine phosphatase that plays a central role in immunity, as demonstrated by the use of calcineurin inhibitors cyclosporine A and tacrolimus (FK506) as immunosuppressants [4]. In the presence of elevated calcium, calmodulin binds to calcineurin, displacing the autoinhibitory domain from the active site, leading to activation of calcineurin and subsequent dephosphorylation of target proteins. Calcineurin substrates include transcription factors, proteins involved in cell cycle and apoptosis, cytoskeletal proteins, scaffold proteins, membrane channels and receptors [5]. The best characterized calcineurin substrates are the nuclear factor of activated T cells (NFAT) transcription factors. Following dephosphorylation by calcineurin, NFAT is translocated to the nucleus where it regulates gene expression. The calcineurin/NFAT pathway is activated in diagnostic breast cancer cases and is essential to survival and metastasis of mammary cancer cells [6]. However, the role of calcineurin/NFAT signaling in ovarian cancer has been unknown.

Recently study revealed that both knockdown and re-expression of NFAT on ovarian cancer cells were employed to observe the effect overgrowth [7]. It was found that NFAT was significantly overexpressed in ovarian cancer tissues in comparison with paired normal control tissues and that overexpression of NFAT was significantly associated with metastasis and poor prognosis on clinical tissue level [7]. Thus, this study aims to investigate whether NFAT upstream protein, calcineurin, affects the prognosis in various histological subtype of ovarian cancer, we have detected the status of calcineurin expression in ovarian cancer tissues and analyzed its clinicopathological significance of calcineurin expression.

## Materials and methods

### Patients

The retrospective study cohort consisted of 50 ovarian cancer patients (19 clear-cell carcinoma, 15 serous carcinoma and 16 papillary serous cystadenocarcinoma) with ovarian cancer who underwent surgical resection at Shengjing Hospital in China between 2007 and 2012. The clinical and pathological characteristics were obtained from patient charts. Tumors were staged according to the Seventh Edition of the Cancer Staging Manual by the American Joint Committee on Cancer, and the histological grade was scored according to the World Health Organization classification. This study was approved by the Ethics Committee of Shengjing Hospital (No.2017PS233K), and written informed consent was obtained from each patient.

### Immunohistochemical staining

Tissues were fixed in formalin and embedded in paraffin, and 2-μm-thick consecutive sections were sliced and mounted on glass slides. The slides were first incubated at 65 °C for 30 min and then subjected to deparaffinization in xylene followed by rehydration in a graded ethanol series. Then, the sections were boiled in Trilogy reagent (Cell Marque, Rocklin, CA, USA) for 10 min for antigen retrieval. After washing with 1 × PBS, the slides were immersed in 3% hydrogen peroxide for 10 min to suppress endogenous peroxidase activity. After three rinses with 1 × PBS, the sections were exposed to a mouse anti-calcineurin antibody (Genetex, Irvine, CA, USA) for 1 h at room temperature. After three rinses with 1 × PBS, the slides were incubated in a biotinylated secondary antibody (Dako, Glostrup, Denmark) for 25 min. The slides were then rinsed three times with 1 × PBS, followed by the addition of horseradish peroxidase (HRP)-conjugated streptavidin for 25 min at room temperature. Peroxidase activity was detected by incubating the slides in the chromogenic substrate 3, 3`-diaminobenzidine (DAB) (Dako) at room temperature. The slides were then counterstained with hematoxylin.

### Statistical analysis

The original IHC data were recorded as continuous variables and were analyzed using Mann-Whitney *U*-test. Kaplan-Meier curve with log-rank test presenting the disease-free survival and overall survival of ovarian cancer exhibiting high or low CN expression. All statistical analyses were performed using SPSS 16.0 and Excel 2007 software. All statistical tests were two-sided, and the thresholds for significance were set at *P* < 0.05 (*).

## Results

### CN is downregulated in later-stage tumors in unique subtype of ovarian cancer

**Fig. 1:**
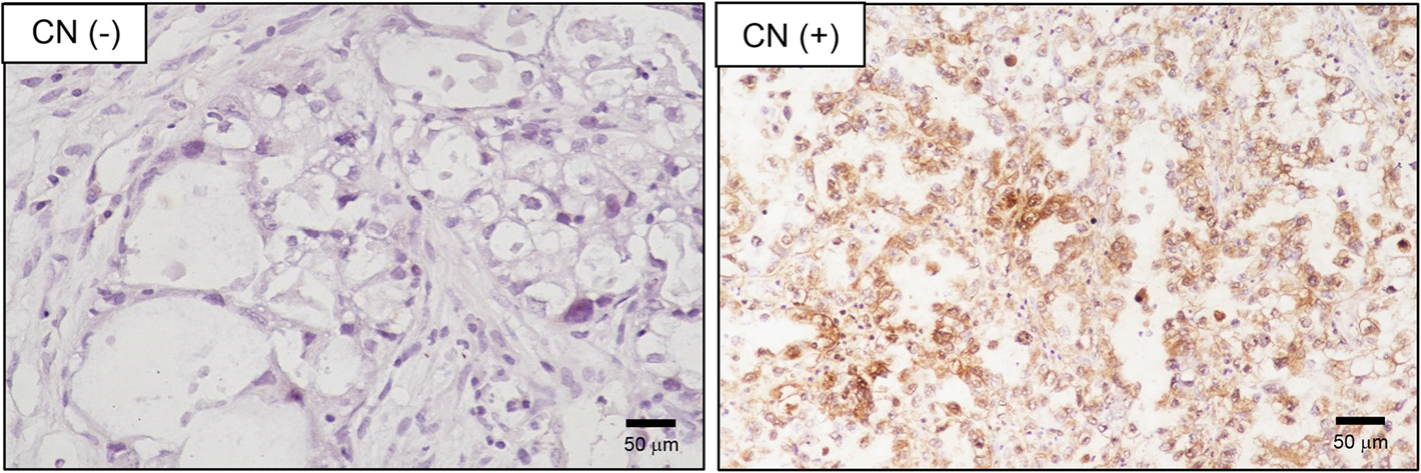
Immunohistochemical staining for CN in ovarian cancer. The tumor tissues of ovarian cancer patients were analyzed for CN expression by immunohistochemical staining as described in *Methods*. CN(−) indicated the CN-negative expression; CN(+) indicated the CN-positive expression. Scale bar: 50 μm

**Fig. 2:**
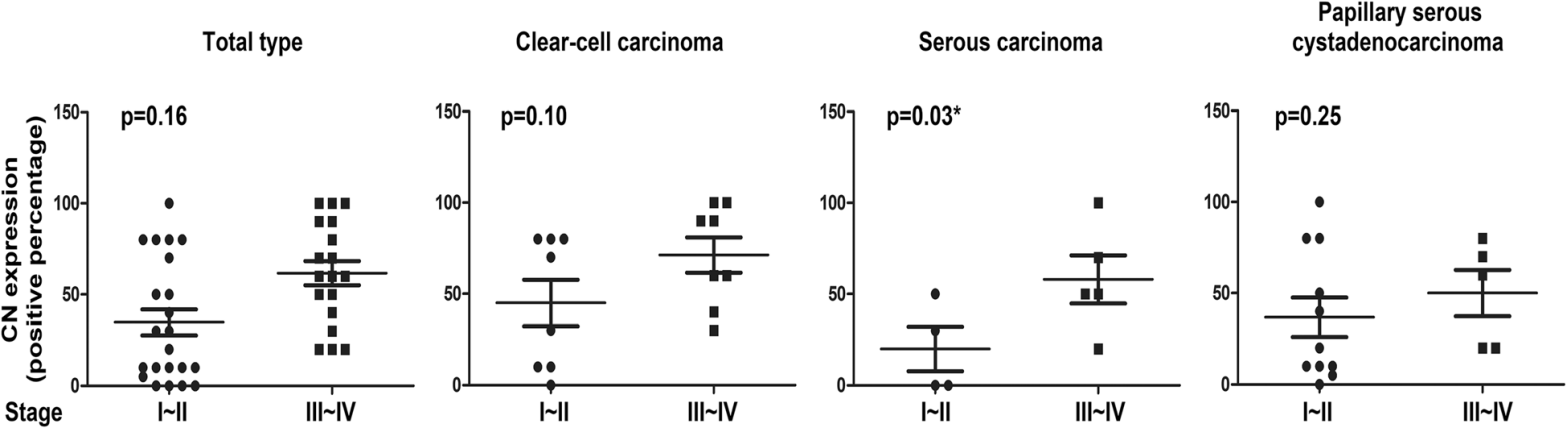
The expression of the CN in ovarian cancer with later-stage or early-stage. The different subtypes of human ovarian cancer, including (**a**) total type, (**b**) clear cell carcinoma, (**c**) serous carcinoma, and (**d**) papillary serous cystadenocarcinoma, were examined the expression of CN via IHC staining (CN-positive percentage). The results were presented as the percentage-change in CN expression of later-stage tumor tissue (stage III~IV) relative to that in early-stage tumor tissue (stage I~II). The CN expression of serous carcinoma was significantly higher in later-stage tumor tissue than in early-stage tumor tissue (*p* < 0.05)

**Fig. 3:**
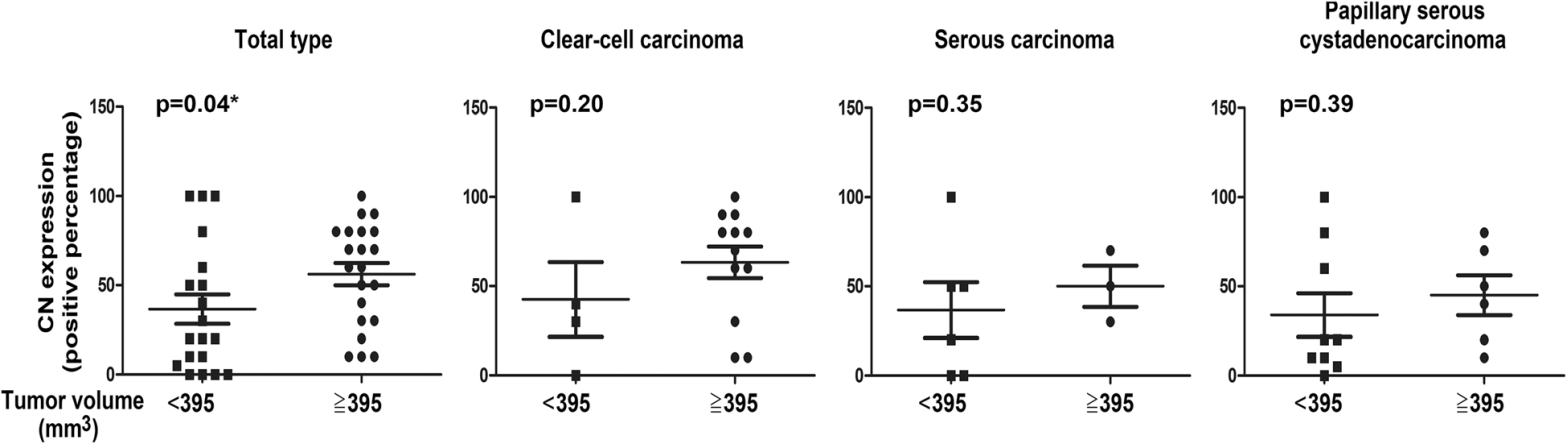
The expression of the CN in ovarian cancer with large or small tumor sizes. The different subtypes of human ovarian cancer, including (**a**) total type (**b**) clear cell carcinoma, (**c**) serous carcinoma, and (**d**) papillary serous cystadenocarcinoma, were examined the CN expression via IHC staining (CN-positive percentage). The results were presented as the percentage-change in CN expression of large tumor size (≧395 mm3) relative to that in small tumor size (< 395 mm3). The CN expression of clear-cell carcinoma, serous carcinoma, or papillary serous cystadenocarcinoma was higher in large tumor size than in small tumor size, but was not statistically significantly (*p* > 0.05)

To analyze the biological significance of CN expression in various histological subtype of ovarian cancer, the levels of CN were analyzed in 19 clear-cell carcinoma, 15 serous carcinoma and 16 papillary serous cystadenocarcinoma via immunohistochemical staining. The CN expression was assessed by the percentage of CN-positive cells in tumor tissues (Fig. 1). The CN expression in different stage of tumor tissue in ovarian cancer is shown in Fig. 2. Compared with early-stage (I~II) tumor tissue, the CN expression was significantly increased in later-stage (III~IV) tumor tissue of serous carcinoma (*p* < 0.05). Further, we evaluated the association of CN expression with tumor size in various histological subtype of ovarian cancer. Consistently, upregulation of CN expression was observed in large tumor size (> 395 mm^3^) of total types (Fig. 3, *p* < 0.05), but was not statistically significantly in large tumor size of each subtype (Fig. 3, *p* > 0.05). The results suggested its potential function as a tumor progressor in ovarian serous carcinoma.

### The upregulation of CN expression is associated with a poor prognosis in unique subtype of ovarian cancer

To determine the clinical significance of CN expression in various histological subtype of ovarian cancer, we performed linear regression analysis using these samples. The examined clinical characteristics of the patients included the following: age, pathological stage, tumor size, lymph node metastasis, distant metastasis, ascites formation, chemotherapeutic response, chemotherapeutic resistance, and the levels of cancer antigen 125 (CA125), cancer antigen 19–9 (CA19–9), cancer antigen 72–4 (CA72–4), alpha-fetoprotein (AFP), and carcinoembryonic antigen (CEA). These characteristics of the recruited ovarian cancer patients are summarized in Table 1. Based on univariate analysis, the CN expression positively correlated with pathological stage in ovarian serous carcinoma, with serum CA125 level in ovarian clear-cell carcinoma, and with serum AFP level in ovarian papillary serous cystadenocarcinoma (Table 1, *p* < 0.05). No significant associations of CN expression with other clinical or pathological parameters were found (Table 1).

**Table 1:** Nonparametric analysis of CN expression in relation to various clinical parameters

Clinical parameters	CN expression											
	Total type			Clear-cell carcinoma			Serous carcinoma			Papillary serous cystadenocarcinoma		
	N	Mean ± SD	*p-*value	N	Mean ± SD	*p-*value	N	Mean ± SD	*p-*value	N	Mean ± SD	*p-*value
Age (years)												
< 50	20	38.25 ± 35.81	0.14	9	51.11 ± 38.87	0.63	3	26.67 ± 25.17	0.38	8	28.13 ± 34.43	0.15
≧50	21	53.33 ± 30.39		8	60.00 ± 31.17		6	48.33 ± 35.50		7	50.00 ± 28.28	
Pathological stage												
I~II	22	31.00 ± 22.73	0.16	8	45.00 ± 21.86	0.10	4	18.00 ± 22.50	0.03*	10	25.00 ± 31.23	0.25
III~IV	18	60.28 ± 33.72		8	71.25 ± 22.48		5	58.00 ± 21.50		5	45.00 ± 32.75	
Tumor size (mm^3^)												
< 395	20	35.25 ± 35.67	0.04*	5	36.00 ± 39.12	0.20	6	36.67 ± 28.30	0.35	9	33.89 ± 36.55	0.39
≧395	21	56.19 ± 28.72		12	63.33 ± 30.85		3	50.00 ± 20.00		6	45.00 ± 27.39	
LN metastasis												
No	24	31.25 ± 31.53	0.26	10	46.00 ± 32.04	0.12	4	20.00 ± 24.50	0.11	10	25.00 ± 31.23	0.25
Yes	17	52.65 ± 36.23		7	68.57 ± 36.25		5	58.00 ± 29.50		5	45.00 ± 32.75	
Distant metastasis												
No	40	40.13 ± 34.02	0.89	16	40.25 ± 35.57	0.68	9	41.11 ± 32.58	─	15	38.33 ± 32.61	─
Yes	1	40 ± 0		1	60 ± 0		0	─		0	─	
Ascites												
No	21	45.24 ± 33.11	0.88	10	52.00 ± 34.90	0.66	6	36.67 ± 38.30	0.55	5	42.00 ± 25.88	0.68
Yes	20	46.75 ± 34.95		7	60.00 ± 36.52		3	50.00 ± 20.00		10	36.50 ± 36.67	
Chemotherapeutic response												
No	2	50.00 ± 56.57	0.91	1	90.00 ± 0	0.32	0	─	─	1	10 ± 0	0.62
Yes	32	47.34 ± 33.41		13	53.85 ± 35.72		7	50.00 ± 31.09		12	38.75 ± 33.04	
Chemotherapeutic resistance												
No	18	35.83 ± 30.01	0.49	5	46.00 ± 28.81	0.81	2	15.00 ± 21.21	─	11	35.00 ± 31.86	0.49
Yes	8	45.00 ± 33.81		4	42.50 ± 35.00		0	─		4	47.50 ± 37.75	
CA125 (U/ml)												
< 324	22	48.86 ± 34.22	0.66	11	51.82 ± 37.10	0.04*	3	50.00 ± 20.00	0.55	8	44.38 ± 37.36	0.61
≧324	18	44.44 ± 33.47		5	72.00 ± 21.68		6	36.67 ± 38.30		7	31.43 ± 27.34	
CA19–9 (U/ml)												
< 21.55	21	50.00 ± 37.68	0.69	8	65.00 ± 34.23	0.90	6	41.67 ± 39.71	1.00	7	40.00 ± 39.58	0.71
≧21.55	17	44.71 ± 28.75		7	50.00 ± 36.52		3	40.00 ± 17.32		7	41.43 ± 26.73	
CA72–4 (U/ml)												
< 7.89	19	47.89 ± 37.94	0.98	8	58.75 ± 39.80	0.83	3	23.33 ± 40.42	0.38	8	46.25 ± 35.43	0.39
≧7.89	21	45.95 ± 29.90		8	57.50 ± 29.16		6	50.00 ± 27.57		7	29.29 ± 28.93	
AFP (ng/mL)												
< 2.9	22	28.86 ± 20.08	0.24	7	57.14 ± 27.52	0.63	3	40.00 ± 36.06	0.91	12	6.67 ± 5.77	0.03*
≧2.9	18	44.44 ± 28.08		9	58.88 ± 39.51		6	41.67 ± 34.30		3	46.25 ± 31.70	
CEA (ng/mL)												
< 1.48	17	48.82 ± 27.36	0.83	8	45.00 ± 34.23	0.14	4	47.50 ± 320.62	0.56	5	56.00 ± 23.02	0.15
≧1.48	22	47.05 ± 38.07		8	71.25 ± 29.49		5	36.00 ± 41.59		9	31.67 ± 35.36	

Statistical analysis using Mann-Whitney *U* test; LN, lymph node; CA125, cancer antigen 125; CA19–9, cancer antigen 19–9; CA72–4, cancer antigen 72–4; AFP, alpha-fetoprotein; CEA, carcinoembryonic antigen; *p < 0.05

**Fig. 4:**
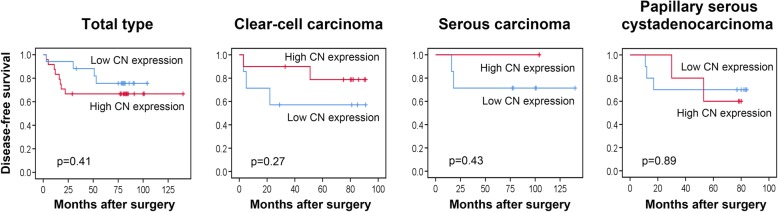
Association of CN expression with disease-free survival in ovarian cancer. The different subtypes of human ovarian cancer, including (**a**) total type, (**b**) clear cell carcinoma, (**c**) serous carcinoma, and (**d**) papillary serous cystadenocarcinoma, were examined the expression of CN via IHC staining (CN-positive percentage). Kaplan-Meier curve presenting the disease-free survival of ovarian cancer exhibiting high or low CN expression. Higher CN expression level (expression level > 50% positive percentage) in tumor tissues of each subtype was not significantly associated with reduced disease-free survival (*p* > 0.05)

**Fig. 5:**
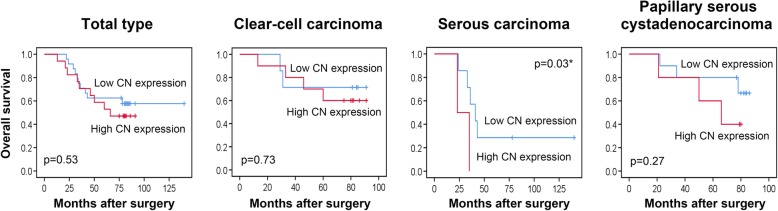
Association of CN expression with overall survival in ovarian cancer. The different subtypes of human ovarian cancer, including (**a**) total type, (**b**) clear cell carcinoma, (**c**) serous carcinoma, and (**d**) papillary serous cystadenocarcinoma, were examined the expression of CN via IHC staining (CN-positive percentage). Kaplan-Meier curve presenting the overall survival of ovarian cancer exhibiting high or low CN expression. Higher CN expression level (expression level > 50% positive percentage) in tumor tissues of serous carcinoma was significantly associated with reduced overall survival (*p* < 0.05)

We further determined whether the CN expression correlated with survival outcomes of patients with ovarian cancer after surgery. Kaplan-Meier survival analysis followed by the log-rank test showed that higher CN expression (expression level > 50%) in tumor tissues not significantly correlated with reduced disease-free survival among patients with each subtype (Fig. 4 and Table 2, *p* > 0.05). Particularly, higher CN expression in tumor tissues significantly correlated with reduced overall survival among patients with serous carcinoma (Fig. 5 and Table 3, *p* < 0.05). The results were further verified by TCGA survival data analysis (http://www.oncolnc.org/). Similarly, a catalytic subunit of CN, *PPP3CA*, is significantly associated with reduced overall survival in ovarian serous carcinoma (Additional file 1: Figure S1, *p* < 0.05). In addition, pathological stage, lymph node metastasis, chemotherapeutic resistance, serum CA72–4 level, serum CEA level and age were identified as significant prognostic factors in ovarian clear-cell carcinoma, serous carcinoma, or papillary serous cystadenocarcinoma, respectively (Table 2 and Table 3, *p* < 0.05).

**Table 2:** Associations between CN expression, clinical parameters and disease-free survival

Clinical parameters	Disease-free survival (months)															
	Total type				Clear-cell carcinoma				Serous carcinoma				Papillary serous cystadenocarcinoma			
	N	Mean	95% CI	*p-*value	N	Mean	95% CI	*p-*value	N	Mean	95% CI	*p-*value	N	Mean	95% CI	*p-*value
CN expression																
≦50	24	97.67	73.67, 121.66	0.41	7	77.70	60.20, 95.20	0.27	7	143.33	92.76, 191.53	0.43	10	62.80	42.71, 82.89	0.89
>50	17	87.15	72.12, 102.19		10	56.29	26.30, 86.27		2	116.39	95.55, 160.14		5	64.60	46.88, 82.32	
Age (years)																
<50	26	181.38	157.41, 205.34	< 0.01*	10	83.33	69.17, 97.50	0.07	7	188.30	163.97, 212.63	0.04*	9	66.71	46.30, 87.12	0.50
≧50	24	82.12	59.05, 105.19		9	51.60	26.31, 76.87		8	98.49	77.65, 142.24		7	57.33	36.48, 78.19	
Pathological stage																
I~II	29	167.83	141.87, 193.79	0.09	9	76.78	57.89, 95.67	0.52	10	168.30	123.97, 212.63	0.99	10	73.40	60.50, 86.30	0.04*
III~IV	20	68.24	48.96, 87.52		9	64.22	39.29, 89.16		5	86.80	56.65, 116.95		6	40.17	14.87, 65.46	
Tumor size (mm^3^)																
< 395	26	132.37	97.50, 167.24	0.19	5	75.60	59.41, 91.78	0.06	12	154.33	103.76, 202.53	0.36	9	65.29	43.36, 87.21	0.08
≧395	24	86.30	72.20, 100.40		14	42.40	7.14, 77.66		3	96.39	75.55, 140.14		7	58.44	38.35, 78.54	
LN metastasis																
No	31	163.70	137.35, 190.06	0.08	11	72.64	54.13, 91.15	0.55	10	168.30	123.97, 212.63	0.99	10	73.40	60.50, 86.30	0.04*
Yes	19	65.44	45.02, 85.86		8	58.75	29.89, 87.61		5	86.80	56.65, 116.95		6	40.17	14.87, 65.46	
Distant metastasis																
No	48	153.55	130.07, 177.03	0.02*	18	69.19	52.48, 85.89	0.17	15	167.80	131.11, 204.49	─	15	64.20	49.30, 79.10	0.15
Yes	2	21.00	19.04, 22.96		1	22.00	22.00, 22.00		0	─	─		1	20.00	20.00, 20.00	
Ascites																
No	26	153.63	121.72, 185.54	0.63	10	66.00	43.09, 88.91	0.98	11	164.33	113.76, 212.53	0.27	5	77.80	66.93, 88.67	0.29
Yes	24	75.63	59.23, 92.02		9	67.44	44.19, 90.70		4	97.49	76.65, 141.24		11	54.00	34.39, 73.61	
Chemotherapeutic response																
No	2	51.00	0, 105.05	0.60	1	82.00	82.00, 82.00	0.48	0	─	─	─	1	20.00	20.00, 20.00	0.07
Yes	37	95.81	77.57, 114.05		15	64.06	41.89, 86.23		10	102.80	70.29, 135.31		12	65.25	49.35, 81.15	
Chemotherapeutic resistance																
No	21	126.95	109.94, 143.96	< 0.01*	5	73.20	50.76, 95.64	0.12	5	141.30	70.29, 212.31	0.02*	11	75.09	63.04, 87.15	< 0.01*
Yes	11	31.95	16.56, 47.33		5	32.60	8.37, 56.83		1	32.00	32.00, 32.00		5	30.20	8.41, 51.99	
CA125 (U/ml)																
< 324	24	133.50	97.04, 169.96	0.18	11	78.46	62.08, 94.83	0.27	5	162.44	111.65, 213.24	0.85	8	71.13	54.12, 88.13	0.28
≧324	24	88.03	75.20, 100.86		7	56.00	26.65, 85.35		9	90.20	66.01, 114.39		8	51.75	29.15, 74.35	
CA19–9 (U/ml)																
< 21.55	24	147.26	112.14, 182.39	0.86	8	71.38	47.59, 95.16	0.71	8	153.29	93.79, 212.78	0.51	8	55.38	35.44, 75.31	0.55
≧21.55	23	105.67	84.02, 127.31		7	66.06	42.89, 89.23		7	124.50	96.08, 152.92		7	64.00	40.54, 87.46	
CA72–4 (U/ml)																
< 7.89	25	174.20	147.38, 201.03	0.04*	8	80.00	59.83, 100.17	0.21	9	164.33	115.76, 212.91	0.80	8	74.75	59.62, 89.88	0.05*
≧7.89	24	69.33	52.59, 86.08		10	60.74	38.11, 83.37		6	89.67	64.02, 115.31		8	47.25	25.78, 76.36	
AFP (ng/mL)																
< 2.9	25	148.04	113.12, 182.97	0.82	7	79.00	66.73, 91.27	0.31	6	183.33	145.14, 221.52	0.30	12	65.25	49.35, 81.15	0.49
≧2.9	24	105.84	86.19, 125.49		11	61.10	37.54, 84.64		9	99.83	57.55, 142.12		4	50.00	16.57, 83. 44	
CEA (ng/mL)																
< 1.48	24	180.33	155.28, 205.39	0.03*	10	81.49	69.17, 97.50	0.02*	8	177.14	128.41, 225.88	0.67	6	58.67	37.69, 79.65	0.39
≧1.48	24	89.07	66.13, 112.02		8	31.60	16.31, 56.87		7	109.38	76.09, 142.66		9	68.44	49.41, 87.48	

Kaplan-Meier test using log rank; LN, lymph node; CA125, cancer antigen 125; CA19–9, cancer antigen 19–9; CA72–4, cancer antigen 72–4; AFP, alpha-fetoprotein; CEA, carcinoembryonic antigen; *p < 0.05

**Table 3:** Associations between CN expression, clinical parameters and overall survival

Clinical parameters	Overall survival (months)															
	Total type				Clear-cell carcinoma				Serous carcinoma				Papillary serous cystadenocarcinoma			
	N	Mean	95%CI	*p-*value	N	Mean	95%CI	*p-*value	N	Mean	95%CI	*p-*value	N	Mean	95%CI	*p-*value
CN																
≦50	24	96.73	76.00, 117.46	0.53	7	73.57	53.15, 93.99	0.73	7	69.29	34.03, 104.54	0.03*	10	73.33	59.06, 87.61	0.27
>50	17	63.24	49.45, 77.02		10	69.8	52.34, 87.26		2	22.00	10.24, 33.76		5	59.4	39.98, 78.82	
Age (years)																
< 50	26	140.79	106.30, 175.28	0.30	10	79.56	65.43, 93.69	0.18	7	118.88	52.82, 184.93	0.63	9	73.44	60.56, 86.33	0.91
≧50	24	86.20	67.27, 105.12		9	60.40	40.08, 80.72		8	72.71	40.43, 105.00		7	63.00	44.19, 81.86	
Pathological stage																
I~II	29	160.80	133.60, 188.00	< 0.01*	9	87.56	81.19, 93.92	0.03*	10	143.50	91.66, 195.34	< 0.01*	10	70.20	56.49, 83.91	0.26
III~IV	20	53.05	40.27, 65.83		9	55.67	34.21, 77.12		5	31.80	24.86, 38.74		6	65.17	46.86, 83.47	
Tumor size (mm^3^)																
< 395	26	123.50	91.08, 155.92	0.71	5	66.60	40.40, 92.80	0.81	12	110.33	61.84, 158.82	0.67	9	71.33	57.58, 85.09	0.98
≧395	24	68.00	56.19, 79.81		14	70.50	55.05, 85.95		3	48.00	22.28, 73.72		7	65.71	47.24, 84.19	
LN metastasis																
No	31	158.09	131.28, 184.90	< 0.01*	11	82.91	72.21, 93.61	0.02*	10	143.50	91.66, 195.34	< 0.01*	10	70.20	56.49, 83.91	0.26
Yes	19	50.95	38.08, 63.82		8	51.00	28.58, 73.43		5	31.80	24.86, 38.74		6	65.17	46.86, 83.47	
Distant metastasis																
No	48	127.95	103.85, 152.05	0.88	18	82.46	76.09, 88.82	0.49	15	106.27	63.19, 149.34	─	15	69.53	57.31, 81.76	0.39
Yes	2	75.00	66.68, 83.32		1	81.00	81.00, 81.00		0	─	─		1	69.00	69.00, 69.00	
Ascites																
No	26	134.35	101.53, 167.16	0.59	10	65.50	49.72, 81.28	0.84	11	113.46	60.45, 166.46	0.65	5	80.40	74.09, 86.71	0.19
Yes	24	66.34	54.61, 78.06		9	70.56	50.00, 91.11		4	55.00	32.34, 77.66		11	63.64	48.48, 78.79	
Chemotherapeutic response																
No	2	66.00	38.28, 93.72	0.99	1	46.00	46.00, 46.00	0.27	0	─	─	─	1	86.00	86.00, 86.00	0.47
Yes	37	91.51	75.18, 107.84		15	70.60	55.21, 85.99		10	66.40	37.16, 95.64		12	85.75	78.54, 92.96	
Chemotherapeutic resistance																
No	21	125.89	110.57, 141.23	< 0.01*	5	88.56	82.19, 94.92	0.01*	5	123.00	87.94, 158.06	0.24	11	77.12	68.46, 85.77	0.02*
Yes	11	52.27	37.78, 66.76		5	49.00	26.58, 71.43		1	59.00	59.00, 59.00		5	49.60	27.15, 72.05	
CA125 (U/ml)																
< 324	24	120.68	85.63, 155.73	0.49	11	77.27	63.36, 91.19	0.41	5	113.89	55.42, 172.36	0.43	8	74.18	59.48, 88.87	0.56
≧324	24	71.67	61.36, 81.87		7	59.86	36.36, 83.36		9	50.40	29.89, 70.90		8	63.88	47.47, 80.28	
CA19–9 (U/ml)																
< 21.55	24	130.61	94.49, 166.73	0.75	8	81.25	63.37, 99.13	0.21	8	134.29	64.96, 203.61	0.22	8	76.88	67.98, 85.77	0.60
≧21.55	23	99.98	80.42, 119.54		9	64.56	47.29, 81.82		7	65.75	34.39, 97.11		7	59.00	37.42, 80.58	
CA72–4 (U/ml)																
< 7.89	25	164.61	134.70, 194.52	< 0.01*	8	86.56	80.19, 92.92	0.01*	9	134.56	76.93, 192.18	0.10	8	69.13	52.32, 85.93	0.26
≧7.89	24	56.17	45.78, 66.56		10	53.67	32.21, 75.12		6	42.83	29.35, 56.31		8	67.88	53.35, 82.40	
AFP (ng/mL)																
< 2.9	25	142.27	108.32, 176.22	0.32	7	73.00	55.03, 90.97	0.60	6	115.00	57.28, 172.72	0.54	12	81.75	74.54, 88.96	0.37
≧2.9	24	87.09	67.74, 106.44		11	66.57	48.66, 84.48		9	70.83	32.42, 109.25		4	64.01	50.29, 77.73	
CEA (ng/mL)																
< 1.48	24	141.13	106.79, 175.46	0.28	10	78.13	62.51, 93.74	0.45	8	132.43	61.69, 203.16	0.28	6	72.33	62.58, 82.09	0.95
≧1.48	24	85.70	65.99, 105.41		8	62.80	44.44, 81.16		7	66.63	36.26, 96.99		9	63.56	45.83, 81.28	

Kaplan-Meier test using log rank; LN, lymph node; CA125, cancer antigen 125; CA19–9, cancer antigen 19–9; CA72–4, cancer antigen 72–4; AFP, alpha-fetoprotein; CEA, carcinoembryonic antigen; *p < 0.05

## Discussion

Ovarian cancer (OC) is one of the most common malignant cancers worldwide and is the tenth leading cause of cancer-related death. Although clinical symptoms are not commonly observed during the early stages of OC development, in most cases, the detection of symptoms during the advanced stage leads to a poor prognosis at the time of diagnosis. Thus, the exploration of new diagnostic and therapeutic molecular targets for ovarian cancer is particularly crucial. In apparent contradiction, activation of calcineurin and its downstream targets also increases tumorigenic potential. As observed by Peuker et al. [8], calcineurin and downstream signalling pathways are activated in colorectal cancer tumors and cell lines, and inhibition of calcineurin decreases cancer stem cell survival and proliferation. Similarly, calcineurin is activated in breast cancer, specifically in triple negative breast cancer, and promotes migration and invasion in vitro and growth and metastasis in vivo [6, 9]. Analogous findings by others support a pro-tumorigenic role for calcineurin signaling in lung, prostate, bladder, pancreatic, and liver cancer, as well as glioblastoma, melanoma and leukemia [10–18]. However, only one report has addressed the function of calcineurin in ovarian cancer. Jin et al. [19] found that Calcineurin B homologous protein isoform 2 (CHP2) was identified to be expressed in ovarian cancer cell line [19]. CHP2-transfected OVCAR3 cells showed increased proliferation rates and exhibited increased activities of cell adhesion, migration and invasion [19]. Further, the present study expanded these studies and identified a potential target, calcineurin, and demonstrated its tumor-progressive effect on ovarian cancer. We found that the calcineurin expression was significantly upregulated in later-stage specimens of ovarian serous carcinoma, associated with serum CA72–4 level in ovarian clear-cell carcinoma, and that higher CN expression in unique subtype of ovarian cancer correlates with a poor prognostic outcome. In addition, pathological stage, lymph node metastasis, chemotherapeutic resistance, serum CA72–4 level, serum CEA level and age were identified as significant prognostic factors in ovarian clear-cell carcinoma, serous carcinoma, or papillary serous cystadenocarcinoma.

The CN/NFAT pathway is activated in diagnostic breast cancer cases and is essential to survival and metastasis of mammary cancer cells [6]. Recently, Xu et al. [20] provided clinical evidence regarding NFAT expression and its clinicopathological significance, finding that overexpression of NFAT in ovarian cancer tissues was significantly associated with metastasis and poor overall prognosis. Mechanistically, it is through activation of ERK1/2/p38/MAPK signaling pathway that NFAT up-regulated the c-myc expression [20]. According to these studies, we further examined whether NFAT-related poor overall prognosis is through its upstream protein CN. However, our data exhibited that NFAT expression was not significantly associated with CN expression in various histological subtype of ovarian cancer (data not shown). Taken together, we suggest that CN-related poor overall survival may be through CN-mediated dephosphorylation of other substrates in ovarian cancer. The substrates of CN, including c-Jun [21], DAXX [22], BAD [23], Rb [24] and Drp1 [25], were associated with the prognosis in ovarian cancer. Further investigations should determine the intrinsic relationship of CN and these downstream proteins on the prognosis of ovarian cancer.

The present study showed that higher CN expression in ovarian serous carcinoma correlates with a poor prognostic outcome. Moreover, the results of TCGA data analysis showed that higher *PPP3CA* expression (a catalytic subunit of the calcineurin) is associated with reduced overall survival in ovarian serous carcinoma. Recent study reported that a novel significantly mutated gene *PPP3CA* in lung adenocarcinoma, but not in lung squamous cell carcinoma [26]. The mutations in *PPP3CA* clustered in the autoinhibitory domain near the C-terminus suggesting it may be gain-of-function alterations [26]. Based on these findings, we suggest that CN-related poor overall survival may be due to the mutation of *PPP3CA* gene in unique subtype of ovarian cancer. Certainly, it merits further investigation.

Carcinoembryonic antigen (CEA) is one of the longest known tumor antigens [27], and at present is still the most widely used tumor marker in the management of colorectal cancer [28, 29]. Elevated serum levels of this assay were demonstrated in patients with ovarian adenocarcinomas, up to 87–88% in mucinous histotypes [30]. Elevation of preoperative serum CEA was also strongly correlated with advanced stage in patients with primary MOC and most likely indicated a poor prognosis [31]. Similarly, cancer antigen 72–4 (CA72–4), a glycoprotein, which increases in gastric, colon, breast, and ovarian adenocarcinomas, may be employed alone or in combination with CA125. CA72–4 is less sensitive than CA125 for EOC, but it is not influenced by pregnancy or the menstrual cycle, and it is only slightly influenced by inflammatory conditions [32, 33]. The present study expanded these findings and further showed that higher serum CEA level is associated with poor disease-free survival in patients with ovarian clear-cell carcinoma. In addition, higher serum CA72–4 level is associated with poor disease-free survival in patients with ovarian papillary serous cystadenocarcinoma and poor overall survival in patients with ovarian clear-cell carcinoma. The results indicate that CEA and CA72–4 as well as CN may be the potential biomarkers for the prediction of prognosis in unique subtype of ovarian cancer.

There are some limitations of the present study. First, this study is the retrospective design, thereby the possibility of residual measured or unmeasured confounding cannot be eliminated, as with any observational investigation. Second, the study was also conducted in a single hospital in an area of China noted for patients diagnosed as ovarian cancer; therefore, the results may not be generalizable to other population. Future studies must broaden the sources of ovarian cancer cases to enhance the reference of research results.

## Conclusion

In the present study, we have, for the first time, found that CN is significantly upregulated in ovarian cancer tissues with later-stage and that the expression of CN, CEA, and CA72–4 was remarkably associated with poor prognosis in unique subtype of ovarian cancer. CN levels may be investigated for use as a prognostic biomarker for risk assessment in unique subtype of ovarian cancer patients.

## Additional file

Additional file 1: **Figure S1.** Association of CN expression with overall survival in ovarian cancer by TCGA data analysis. A pan-cancer analysis using data in OncoLnc, which linked TCGA survival data to mRNA. Kaplan-Meier curve presenting the overall survival of ovarian cancer exhibiting high or low *PPP3CA* (a catalytic subunit of CN) expression. Higher *PPP3CA* expression (expression level > 50%) in the tumor tissues of serous carcinoma was significantly associated with reduced overall survival (*p* < 0.05). (TIF 3186 kb)

## Data Availability

The datasets used and/or analyzed during the current study are available from the corresponding author on reasonable request.

## Abbreviations

AFP: alpha-fetoprotein
CA125: cancer antigen 125
CA19–9: cancer antigen 19–9
CA72–4: cancer antigen 72–4
CEA: carcinoembryonic antigen
CN: calcineurin
IHC: immunohistochemistry
OC: ovarian cancer
