# Supplementary figures and images for: Higher expression of calcineurin predicts poor prognosis in unique subtype of ovarian cancer
Source: J Ovarian Res. 2019 Aug 9;12:75. doi: 10.1186/s13048-019-0550-0 (PMC6688357; doi:10.1186/s13048-019-0550-0)

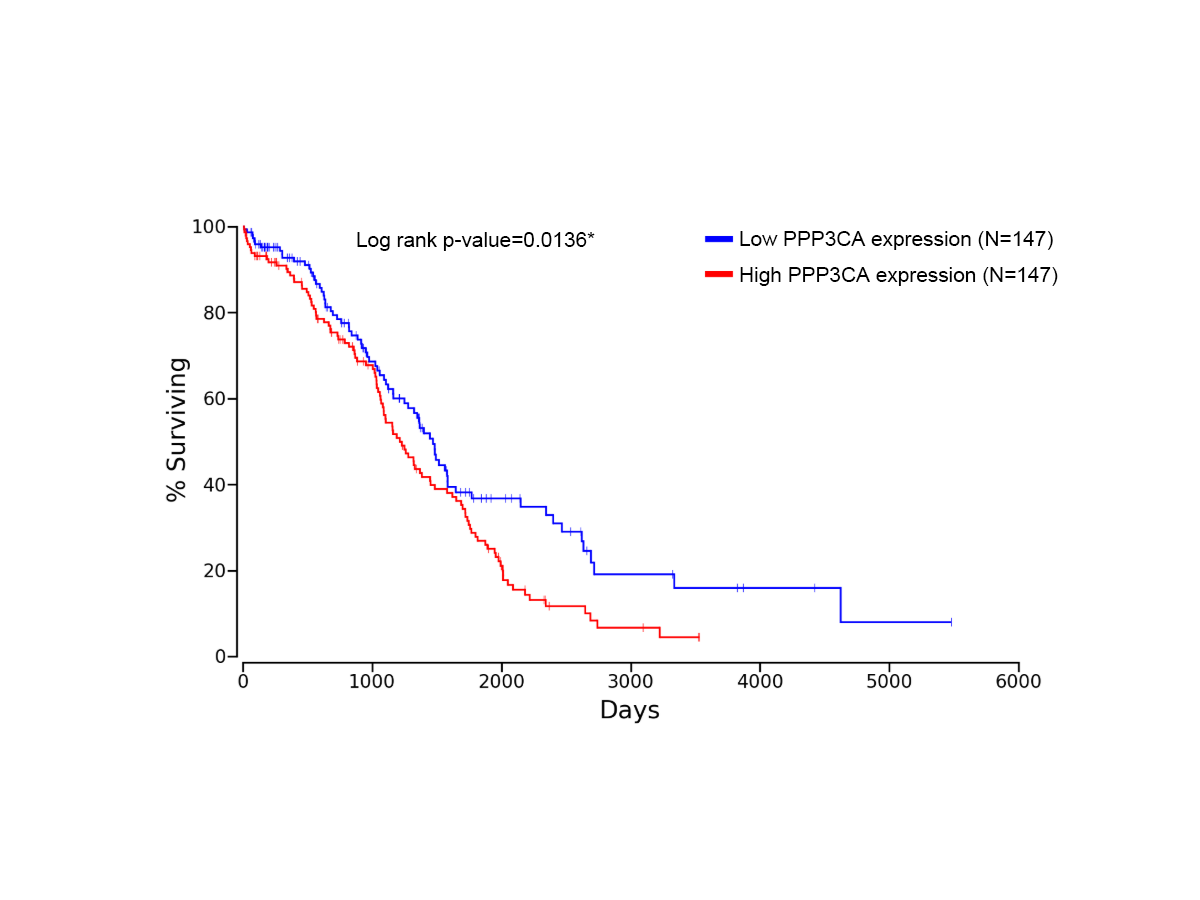

Supplement: Supplementary file 1 — Figure S1. Association of CN expression with overall survival in ovarian cancer by TCGA data analysis. A pan-cancer analysis using data in OncoLnc, which linked TCGA survival data to mRNA. Kaplan-Meier curve presenting the overall survival of ovarian cancer exhibiting high or low PPP3CA (a catalytic subunit of CN) expression. Higher PPP3CA expression (expression level > 50%) in the tumor tissues of serous carcinoma was significantly associated with reduced overall survival (p < 0.05). (TIF 3186 kb) [file 13048_2019_550_MOESM1_ESM.tif]
